# Mechanical analysis of femoral neck fracture fixation with dynamic condylar screw in synthetic bone

**DOI:** 10.1590/1413-78522014220500922

**Published:** 2014

**Authors:** Anderson Freitas, Rafael Almeida Maciel, Renato De Almeida Lima, Diogo Ranier De Macedo Souto, Marcelo De Almeida Ferrer

**Affiliations:** 1 Hospital Ortopédico e Medicina Especializada, Brasília, DF, Brazil, Hospital Ortopédico e Medicina Especializada (HOME), Brasília, DF, Brazil

**Keywords:** Femoral neck fractures, Biomechanical phenomena, Internal fixators

## Abstract

**Objective:**

To analyze statistically results in biomechanical testing of fixation of femoral neck Pauwels type III fractures, on synthetic bone, with dynamic condylar screw (DCS) and control group.

**Methods:**

Ten synthetic bones of a national brand were used. Test Group: fixation was performed after osteotomy at 70^o^ tilt using DCS plate with four holes. We analyzed the resistance of this fixation with 5 mm displacement and rotational deviation (Step 1) and with10 mm (Step 2). Control group: the models were tested in their integrity until the femoral neck fracture occurred.

**Results:**

The values of the test group in Step 1 showed a mean of 974N and SD = 114N. In Stage 2, we obtained on average 1335N and SD = 98N. The values in the control group were: 1544N, 1110N, 1359N, 1194N, 1437N, respectively. Statistical analysis using the Mann-Whitney test for comparison of the maximum force (N) between the test group and the control, in Step 2, demonstrated that there is no significant difference between the DCS and control plates (p = 0.91).

**Conclusion:**

There is no significant difference between the DCS boards and the control group exposed to full resistance.* Level of Evidence III, Case Control.*

## INTRODUCTION

With increasing life expectancy and the growing number of high-energy trauma, fractures of the femoral neck are becoming a surgical entity increasingly frequent.^1^^,^ 2 These fractures have a bimodal characteristics: young patients, victims of high energy trauma, and the elderly, which accounts to most of the injuries. According to the World Health Organization (WHO), it is estimated that in 2050 6.3 million fractures will occur in the proximal extremity of the femur, a three higher figure than the current one.^1^^,^ 2

Femoral neck fractures can be classified according to the following criteria: AO group, Garden, and Pauwels.^1^^,^ 3 Pauwels classification can be useful in femoral neck fractures in young adults since they describe the orientation of the line fracture - obliquity angle of the fracture line in relation to the horizontal, as in AP radiography.^4^ Fractures type I show angle lower than 30 degree; Type II shows the fracture line between 30 and 50 degrees; Type III have an angle larger than 50 degrees.^5^^,^ 6 Given the increase in the number of high-energy trauma in young patients, femoral neck fractures Pauwels type III, the injury pattern in this population, are becoming increasingly frequent, acquiring importance in the health context of our society.^1^^,^ 7

Treatment of femoral neck fracture is defined based on the fracture pattern, bone quality, comorbidities, and physiological age of patients.^1^^,^ 8 However, there is no doubt about the benefit of surgical treatment, reducing the rate of morbidity and mortality.^9^ Such treatments are defined as anatomical reduction with fracture fixation or arthroplasty.^1^^,^ 10 Several methodologies of internal fixation have been used with clinical and biomechanical variables results in several studies. The main methods are dynamic hip screw (DHS) and cannulated screws in different assemblies.

A recent retrospective clinical study confirmed better rates of union of vertical fractures of the femoral neck when treated with fixed angle devices, compared with cannulated screws.^4^ Biomechanical studies have shown that DHS associated with anti-rotational screws were better than cannulated screws.^11^^,^ 12

The dynamic condylar screw (DCS) was originally designed for use in fractures of the distal femur and intercondylar fractures, but has found increasing application in proximal femoral fractures, particularly subtrochanteric ones. This device has been studied and compared with cannulated screws and fixation with DHS showing inconclusive results. Liporace et al.^4^ specifically examined the Pauwels type III fractures and found a trend toward lower failure of fixation with a fixed angle device (DHS cephalomedullary rod or DCS), compared with cannulated screws. Aminian *et al*.^13^ also compared four fixation buildings in fractures Pauwels III simulated in samples of fresh frozen cadavers. The study showed a superior strength of the proximal femoral locking plate (PFLP) compared with DHS, DCS, and 7.3mm cannulated screws. The authors mentioned that, although the PFLP was the most rigid construction, the implant does not allow compression at the fracture site and can, therefore, affect healing *in vivo*.^13^ Since the fractures Pauwels type III are fractures with angulation greater than 50 degrees and it is subjected to strong vertical shear force, we decided to evaluate the use of DCS in fractures Pauwels type III.

The femoral neck fractures Pauwels III are related to a higher rate of complications as a result of shear forces and varus instability, leading to failure in fixation, non-union, and osteonecrosis.^13^^,^ 14 Thus, there is greater interest to evaluate the best fixation way in order to avoid the inherent complications of this fracture pattern.

The authors propose a mechanical analysis of DCS fixation for femoral neck fractures Pauwels type III, compared with the control group, with the objective of determining the effectiveness of this fixation.

## MATERIALS AND METHODS

Ten synthetic bones of a national brand, developed in rigid polyurethane for cortical layer and trabecular to the spongy part model C1010 were used, divided in two groups, control and test.

All samples were pre-perforated for the initial placement of the implant under fluoroscopic guidance before the osteotomy to facilitate anatomic reduction and optimum positioning of the implant.

A vertical fracture of the femoral neck was made with a band saw at a 70 degree angle to the horizontal axis simulating a Pauwels type III fracture. The osteotomy was performed with a pre-made template for which there were no angular difference between the tested bones.

**Figure 1:**
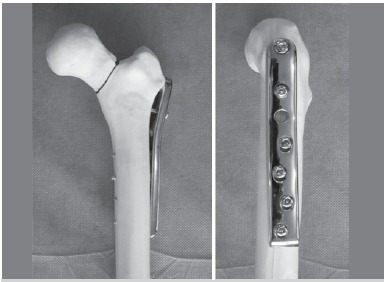
Fixation with DCS pre-test.

**Figure 2:**
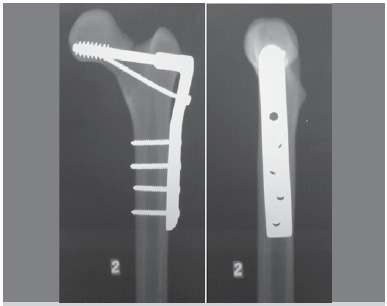
Pre-test X-Ray.

The fixation of the five bones of the test group were performed with DCS with four holes using the 95° guide, establishing it as a reference for placing the 90 mm sliding bolt , at a point 25mm below the apex of the greater trochanter in the middle -lateral position. The screw tip was positioned 5 to 10 mm of the subcondral bone. The plate was fixed to the femoral shaft with four 4.5 mm cortical screws. This system has been blocked with cotter pin, compressing the osteotomy focus. We used a 100 mm anti-rotatory screw through the proximal opening of plate crossing posteriorly and inferiorly the sliding pin. For correct positioning, fluoroscopic control was performed in AP and profile view during each step of the procedure. (Figure 1) Following the procedure, assemblies were subjected to X-ray for reduction evaluation and proper positioning of the synthesis. (Figure 2)

**Figure 3:**
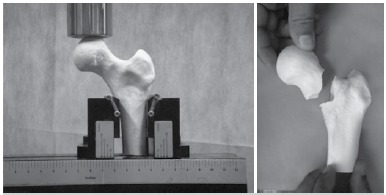
Control group.

The remaining five bones, identified as a control group were tested with their integrity intact until the fracture of the femoral neck occurred, simulating, in this way, the resistance maximum load previous to the intact synthetic bone femoral neck. It has been, thus, defined, the maximum load prior to fracture occurrence and the comparison parameter to the need for resistance to the synthesis method used in the test group. (Figure 3)

### Test group

**Figure 4:**
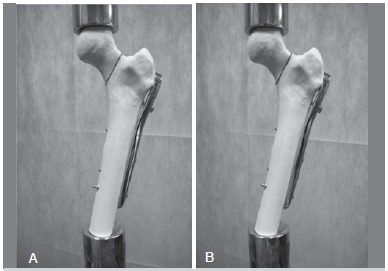
A) Image of bone model fixated at the testing machine at pre-test; B) Image of bone model fixated at the testing machine during the 5mm displacement assay (Step 1).

**Figure 5:**
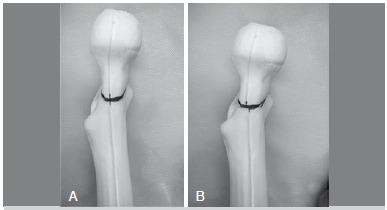
A) Marking to assess rotational deviation pre-test; B) Image of misaligned mark after Step 1 assay on test group.

**Figure 6:**
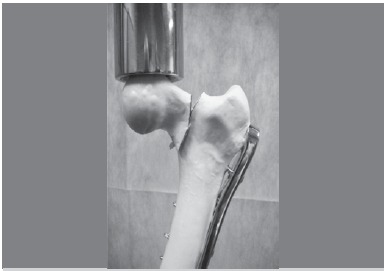
Image of the bone model used at the 10mm displacement assay (Step 2).

Fixed synthetic femurs had 200 mm in length, and were positioned vertically with an inclination of 25 degrees in valgus. (Figure 4) The load application system transmitted force to the apex of the femoral head leading to an incremental loading of strength and load to failure. The analysis of the mechanical testing of this group was divided into two stages: the fixation strength within 5mm displacement (Figure 4B) and evaluation of rotational deviation. (Figure 5) (Step 1), and fixation strength in 10 mm displacement (Step 2). (Figure 6)

With this format of essay, the applied force tested the strength of the synthesis assembly on the osteotomy focus.

### Control group

Not fixated synthetic femurs (control group) had a 125 mm length, and were positioned vertically in neutral tilt. The load application system transmitted strength to the apex of the femoral head, this has been applied until there was a fracture of the femoral neck (Figure 3) simulating, therefore, the maximum pre-fracture resistance.

**Figure 7:**
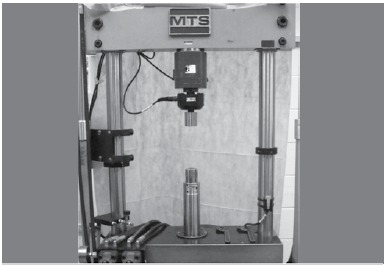
Test machine used on the assays.

We used a load application speed of 20 mm/ min in the testing machine MTS (Materials Testing System) Model 810 - FlexTest 40, with capacity of 100kN. In the test a load cell with 10kN capacity calibrated and tested was used. The axial force was applied to the femoral head through engagement with the piston surface of the equipment. (Figure 7)

### Statistical analysis

The statistical method used was the Mann-Whitney test for comparison of the maximum force (N) between two types of plates. Nonparametric method was used because the maximum force is not normally distributed (Gaussian distribution) due to the small sample size analyzed in each type of plate.

The criterion for determining significance was set at 5%. Statistical analysis was performed with (SAS 6:11 software, SAS Institute, Inc., Cary, North Carolina, USA).

In this analysis of mechanical fixation of fractures of the femoral neck, as it is not a clinical trial, in which no medication of any type was used, either human or animal tissue, we did not submit the essay to the Ethics Committee.

## RESULTS

### Test group

**Figure 8:**
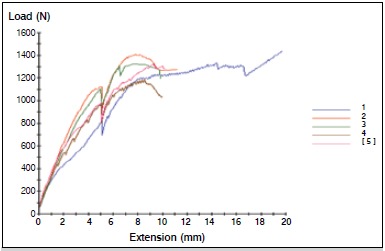
Strength x displacement curves.

The load value in Newtons (N) applied until the fracture displacement of 5 mm was 809, 1119, 1025, 936, and 983, respectively, for samples 1 through 5, which presented as average value of 974N and standard deviation of 114N. The maximum loads in Newtons (N) applied to five samples were:1438, 1409, 1323, 1186, and 1321, respectively. They presented as mean the value of 1335N and standard deviation 98N. The load in Newtons (N) applied to cause rotational deviation of the femur with 5 mm displacement of the head fracture in the five samples were 0.1; 0.0; 0.0; 1.7; and 0.3 respectively, presenting mean of 0.42N and standard deviation of 0.72N; as shown in Table 1 and Figure 8.

**Table 1:** Load values in Newtons (N) in 5 mm and 10 mm displacement and rotational deviation (degrees).

**Sample**	**Load in 5mm displacement (N)**	**Load in 10mm displacement (N)**	**Rotation (degrees)**
1	809	1438	0.1
2	1119	1409	0
3	1025	1323	0
4	936	1186	1.7
5	983	1321	0.3
Mean	974	1335	0.42
Standard deviation	114	98	0.72

### Control group

**Figure 9:**
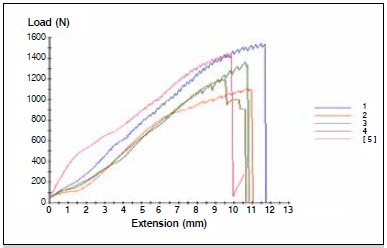
Strength x displacement curves for the control group.

The value of maximum load in Newtons (N) in the five samples from the control group were respectively 1544, 1110, 1359, 1194, and 1437N, showing as an average value of 1329N and standard deviation 177N (Table 2 and Figure 9)

**Table 2:** Maximum load (N) in the control group.

**Sample**	**Maximum load (N)**
1	1544
2	1110
3	1359
4	1194
5	1437
Mean	1329
Standard deviation	177

**Figure 10:**
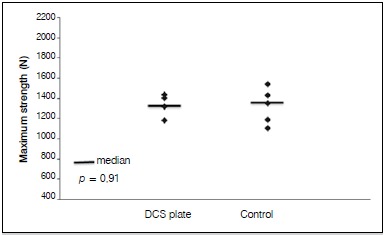
Maximum strength at 10 mm displacement for DCS plate and control.

According to the Mann-Whitney test, it was observed that there was no significant difference in the maximum strength in 10 mm displacement between the plates and DCS control (p = 0.91). (Figure 10)

## DISCUSSION

The ideal surgical fixation for femoral neck fracture should resist the weight-bearing forces and restrict movement across the fracture site during bone healing, allowing a quick and safe recovery of the patient and his return to daily activities. Secure attachment also reduces the high complication rates reported for the treatment of this injury.^15^

During daily activities, the load on the femoral head is alternated anteriorly and posteriorly, determining varus forces, and in the presence of fractures, vertical shear forces. The force applied to the femoral head-neck depends on the patient's weight and the activity performed, and this will be of fundamental importance in the resistance of the implant in femoral neck fractures. We used as reference in this study an axial force of 1400N as the force applied to the hip of a 70 kg person supported on one leg.^16^

To date there are few biomechanical studies using DCS in fractures of the femoral neck. Aminian *et al*.^13^ conducted a study comparing four methods of fixation of Pauwels III fractures, demonstrating the superiority of material resistance to axial force applied to the assembly. In decreasing order of stability, resisting to greatest strength, are the fixed angle locked plate, then the DCS, DHS and three cannulated screws. Another biomechanical study was performed by Sirkin *et al.*,^17^ demonstrating a better fixation of femoral neck fractures oriented vertically in cadaver bones, using a cross-bolt at the calcar and two parallel cancellous screws (called XCS) or DCS compared to DHS or three parallel cannulated screws.^17^

This biomechanical study demonstrates that using DCS for fixation of femoral neck Pauwels type III fracture in synthetic bone undergoes a displacement of 5 mm, i.e., starts its synthesis loss with the application of an average load of 974N associated with a rotation of 1.37 degrees thus, supporting, in these samples, a maximum load of 1335N on average, statistically similar to a femur with no fracture (control group with maximum load 1329N). Thus, it is believed that the use of this method of fixation of these fractures brings adequate stability.

We recognize the limitations of this study using synthetic bone rather than cadavers bones used in the studies by Aminian

*et al.* 13 and Sirkin *et al*.,^17^ since the former do not correctly reflect the anatomy of the femoral trabecular bone and its supporting force. We did not simulate all physiological components of the force - cyclic, torsional, axial - to which the hip is subjected during ambulation or muscle contraction alone. Directional vector forces could have resulted from changes in the load values and, consequently, stabilize the implant. The axial load in one single direction does not simulate the complex system of loads applied to the hip during walking, as well as the torsional forces and the orientation of the vectors that change during the hip movements. However, all of the shortcomings of this study probably originate quantitative differences (level of applied force), rather than qualitative ones. Thus, they do not compromise the validity of the study.

The choice of synthetic bone was determined to ensure comparable biomechanical properties between the groups, thus, eliminating variables.^18^ Therefore, we eliminate potential changes inherent to the human bones which make, due to their non-uniform characteristics (bone density, diameter and length), the evaluation of the fixation method questionable, determining only the stiffness assay of the implant.

The results of this biomechanical analysis confirm the importance of the fracture pattern and the possibility of using the DCS for surgical fixation, despite its design and purpose have not been developed for it. Enhancing the mechanical angular disposition of the implant to the fracture site, thereby determining the possibility of developing new implants with appropriate principles and designs.

## CONCLUSION

There is no significant difference between the DCS plates and the control group exposed to full strength, establishing a possibility of using DCS in femoral neck fractures.
